# Prevalence of antimicrobial resistance and potential pathogenicity, and possible spread of third generation cephalosporin resistance, in *Escherichia coli* isolated from healthy chicken farms in the region of Dakar, Senegal

**DOI:** 10.1371/journal.pone.0214304

**Published:** 2019-03-26

**Authors:** Passoret Vounba, Julie Arsenault, Rianatou Bada-Alambédji, John M. Fairbrother

**Affiliations:** 1 Department of Pathology and Microbiology, Faculty of Veterinary Medicine, Université de Montréal, The Swine and Poultry Infectious Diseases Research Centre (CRIPA) and the Research Group on Zoonoses and Public Health (GREZOSP), St-Hyacinthe, Quebec, Canada; 2 Department of Public Health and Environment, Ecole Inter-Etats des Sciences et Médecine Vétérinaires (EISMV) de Dakar, Dakar, Senegal; Nitte University, INDIA

## Abstract

*Escherichia coli* is a normal inhabitant of the intestinal microbiota of chickens, a small proportion of which may be avian pathogenic *E*. *coli* (APEC) or potential extraintestinal pathogenic *E*. *coli* (ExPEC), capable of causing disease in humans. These *E*. *coli* may also be resistant to antimicrobials of critical importance in human or veterinary health. This study aims to 1) determine the prevalence of antimicrobial resistance (AMR) and resistance genes, multidrug resistance (MDR), chromosomal mechanisms of quinolone-resistance and virulence profiles of *E*. *coli* isolated from healthy chicken farms in the region of Dakar, Senegal, 2) investigate the spread of third-generation cephalosporins (3GC) resistance in *E*. *coli* isolated from healthy chicken farms with respect to virulence and resistance genes, serogroups, Pulsed-Field Gel Electrophoresis (PFGE), phylogenetic groups, plasmid types and transferability and 3) determine whether nonsusceptibility against 3GC on farms could be linked to risk factors. More than 68% of isolates from environmental faecal and drinking water samples, carcasses and carcass washes collected on 32 healthy chicken farms were multidrug resistant (MDR), resistance to antimicrobials critical in human health (3GC or ciprofloxacin) being found in all types of samples. Ciprofloxacin resistance was due to mutations in the *gyrA* and *parC* genes, 95% of tested farms harboring isolates carrying three mutations, in *gyrA* (Ser83Ile and Asp87Asn) and *parC* (Ser80Ile). Nine of the 32 farms (28.1%) demonstrated the presence of one or more 3GC-nonsusceptible indicator isolates but none of the potential risk factors were significantly associated with this presence on farms. Following ceftriaxone enrichment, presumptive extended-spectrum beta-lactamase/AmpC-beta-lactamase (ESBL/AmpC)-producer isolates were found in 17 of the 32 farms. 3GC resistance was mediated by *bla*_*CMY-2*_ or *bla*_*CTX-M*_ genes, *bla*_*CTX-M*_ being of genotypes *bla*_*CTX-M-1*_, *bla*_*CTX-M-8*_ and for the first time in chickens in Senegal, the genotype *bla*_*CTX-M-15*._ Clonally related ESBL/AmpC-producer isolates were found on different farms. In addition, *bla*_*CTX-M*_ genes were identified on replicon plasmids I1 and K/B and *bla*_*CMY-2*_ on K/B, I1 and B/O. These plasmids were found in isolates of different clusters. In addition, 18 isolates, some of which were ESBL/AmpC-producers, were defined as potential human ExPEC. In conclusion, *E*. *coli* isolates potentially pathogenic for humans and demonstrating MDR, with resistance expressed against antimicrobials of critical importance in human health were found in healthy chickens in Senegal. Our results suggest that both clonal spreading and horizontal gene transfer play a role in the spread of 3GC-resistance and that chickens in Senegal could be a reservoir for AMR and ExPEC for humans. These results highlight the importance of raising awareness about compliance with biosecurity measures and prudent use of antimicrobials.

## Introduction

*Escherichia coli* is a normal inhabitant of intestinal flora of warm-blooded animals including humans, but may also be a potential pathogenic agent. In birds, pathogenic strains are termed avian pathogenic *E*. *coli* (APEC), a sub-group of the pathotype of Extraintestinal pathogenic *E*. *coli* (ExPEC). APEC cause avian colibacillosis, often following a weakening of the immune defences and leading to significant economic losses [1]. *E*. *coli* isolates may be classified into four phylogroups A, B1, B2 and D. Isolates from phylogroups B2 and D possess more virulence genes associated with ExPEC than those of phylogroups A and B1 [2]. Several studies have demonstrated a genetic similarity between *E*. *coli* involved in urinary tract infections, meningitis, and septicaemia in humans and those found in poultry and poultry products for human consumption [3–6], suggesting that the chicken could act as a reservoir of human pathogenic *E*. *coli*.

Antimicrobial use has increased the life expectancy of humans and greatly improved the productivity of animals. However, these benefits have been compromised by the development of antimicrobial resistance (AMR) [7]. Antimicrobial agents of critical importance in human health (e.g., ceftriaxone and ciprofloxacin) and animal health (e.g., ceftiofur) are not exempt from this phenomenon. *E*. *coli* resistance against ceftriaxone is mediated by the *bla*_*CTX-M*_ gene encoding production of Extended-spectrum β-lactamases (ESBLs) and ceftiofur resistance is mediated by the *bla*_*CMY*_ gene encoding class C β-lactamases (AmpC). ESBL/AmpC-producing *E*. *coli* often also demonstrate resistance to a wide range of antimicrobials including fluoroquinolones, trimethoprim-sulfamethoxazole and tetracyclines [8, 9].

ESBL/AmpC genes carried by *E*. *coli* isolates are located on incompatibility plasmid groups such as I1, N, A/C, P and I1 [10, 11], often together with genes for resistance to other antimicrobials, resulting in multidrug resistance (MDR) and the possibility of a transfer to other bacterial species, including those pathogenic for humans. In addition, because of the co-existence of some virulence genes on the same replicon plasmids, some studies suggest that there may be an association between AMR and virulence [12, 13]. *E*. *coli* resistance to fluoroquinolones such as ciprofloxacin may involve plasmid genes such as *qnr* genes, but the most important mechanism is acquisition of mutations in quinolone resistance-determining regions (QRDR) of *gyrA* and *parC* genes [14] and this mutational quinolone resistance may be clonally spread as it has been reported in *E*. *coli* of sequence type 131 (ST131) [15].

Certain risk factors may be associated with the presence on farms of *E*. *coli* isolates resistant to antimicrobials critically important for humans. A study in Canada found that *in ovo* administration of ceftiofur and the use of hydrogen peroxide to disinfect water lines during the growing period was associated with an increase in the prevalence of *E*. *coli* isolates simultaneously resistant to amoxicillin/clavulanic acid, ceftiofur and cefoxitin [16]. In Norway, the risk of detection of cephalosporin-resistant *E*. *coli* in chickens was reported to be related to the status of the previous flock in the broiler house, the number of parent flocks supplying the broiler flock with day-old chickens, and transport personnel entering the room where broilers are raised [17]. Other factors that may promote the introduction of new clones in a farm are the use of contaminated well water as drinking water and the presence of insects or rodents which may serve as mechanical vectors [18, 19].

The use of 3^rd^ generation cephalosporins (3GC) in chickens in Senegal has not yet been reported, although this practice cannot be excluded because antimicrobials can be purchased by farmers from street vendors [20]. In addition, we recently found that multiresistant *E*. *coli* isolated from chickens with colibacillosis in Senegal carried ExPEC-associated virulence genes and some isolates were carriers of *bla*_*CTX-M-1*_ or *bla*_*CTX-M-8*_ [21].

We hypothesized that potential human ExPEC and 3GC resistant pathogenic *E*. *coli* are present in healthy chickens in Senegal and that the presence of the latter strains is associated with some farm characteristics such as antimicrobial use, management practices or on-farm biosecurity. The objectives of this study were to 1) Determine the prevalence of antimicrobial resistance and resistance genes, multidrug resistance (MDR), chromosomal mechanisms of quinolone-resistance and virulence profiles of *E*. *coli* isolated from healthy chicken farms in the region of Dakar, Senegal at both isolate and farm levels, 2) investigate the spread of 3GC nonsusceptibility in *E*. *coli* isolated from healthy chicken farms with respect to virulence and resistance genes, serogroups, PFGE, phylogenetic groups, plasmid types and transferability and 3) determine whether nonsusceptibility against 3GC on farms could be linked to risk factors.

## Material and methods

### Sample and data collection

A cross-sectional study was conducted on private poultry farms located in the area of Dakar, Senegal. A list of farms present in the study area was provided by the Senegal Livestock Department and was updated with private veterinarians in the study area. A total of 68 farms located in 17 boroughs around the city of Dakar were identified. These farms belong to the three commercial poultry farming sectors [20]: sector 1, integrated poultry farming with 3000 to 18000 chickens; sector 2 with 1000 to 2000 poultry head; and sector 3 with small livestock farms of 50 to 500 chickens, often integrated into residential homes.

Based on the population size of 68 farms, an expected prevalence of farms with 3GC-nonsusceptible isolates of 50%, a precision of 12% and a confidence level of 95%, the required sample size for prevalence estimation at the farm level was estimated as 34 farms using the WinEpiscope 2.0. This 50% expected prevalence was used as a conservative approach as no studies had previously estimated the prevalence of farms harbouring *E*. *coli* potentially pathogenic for humans and/or producers of ESBL/AmpC in Senegal. A proportional stratified sampling method was then used to select the 34 farms, with each stratum representing 4 to 5 rural communities grouped according to their geographical proximity.

When a farm consisted of one chicken house, samples were taken from this house whereas, when there were at least two houses, samples were collected in two separate houses. In each house, one sample of fresh faeces and one of drinking water were collected between May and July 2011. Each sample of faeces or drinking water consisted of a pool of five samples taken in different parts of the house or in different troughs. In addition, one rinsing water (water in which the last rinsing of the carcasses was carried out) and one carcass swab sample were collected when slaughter of birds was carried out on the farm. Since the carcasses are stacked one on top of the other following plucking, evisceration and rinsing, several carcasses of the stack were wiped with a single cotton swab.

The collected samples were transported in a cooler to the microbiology laboratory of the École Inter-États des Sciences et Médecine Vétérinaires (EISMV) of Dakar where they were analyzed. In addition, a questionnaire developed by our team (available in French on request) was completed by each farmer at the time of sampling, to collect data relating to biosecurity measures and use of antimicrobial agents on the farm.

### Isolation of *E*. *coli* and establishment of *E*. *coli* collections

Each pooled faecal sample was homogenised 1/10 (weight/volume) in buffered peptone water and filtered using a Whirl-pak sterile sample bag with pore size of 0.45 **μ**m. The filtrate was streaked on MacConkey plates and incubated overnight at 37°C. For drinking water and rinsing water, 250 ml of each sample was centrifuged at 8000 rpm for 15 min; the pellet was seeded on MacConkey agar and incubated overnight at 37°C. Carcass swabs were directly streaked on MacConkey agar. All primary cultures were kept at 4°C until shipping to the *Escherichia coli* reference laboratory (EcL, Université de Montréal, Canada).

#### Indicator *Escherichia coli*

One (or two morphologically different colonies when detected) lactose-positive isolates on MacConkey agar, that were positive for the *uidA* gene encoding β-glucuronidase, were selected. PCR conditions used to detect *uidA* gene included initial denaturation (95°C, 2 mn), 24 cycles of denaturation (94°C, 30 s), annealing (65°C, 30 s), extension (72°C, 30 s), and final extension (4°C).

#### Specific collections

**Potential ExPEC:** Boiled DNA of a pool of five lactose-positive colonies per sample, selected from colonies not included in the indicator *E*. *coli* collection, was examined by PCR [22] for detection of ExPEC-associated virulence genes *iucD*, *tsh*, *cnf* and *papC* permitting a rapid and inexpensive initial screen for a wide spectrum of possible ExPEC strains [23] including APEC considered to have zoonotic potential [6]. When the pool of five colonies was positive, each isolate was individually tested for the four ExPEC-associated virulence genes, and then one isolate per sample was randomly selected for each virulence profile and examined by PCR for additional virulence genes associated with ExPEC or APEC [21].

**Potential ESBL/AmpC-producers:** We used the protocol described previously by Agersø and colleagues [24] with some modifications. Briefly, 50 μl of the culture stored at -80°C was inoculated in 5 ml of peptone water. After 30 minutes of incubation at 37°C, 20 μl of the broth was streaked on MacConkey agar supplemented with 1 mg/L of ceftriaxone. After overnight incubation at 37°C, five lactose-positive colonies per sample, when available, were selected and confirmed as *E*. *coli* by the presence of the *uidA* gene.

### Antimicrobial susceptibility testing

All indicator *E*. *coli*, potential ExPEC and potential ESBL/AmpC isolates were tested against 14 antimicrobials belonging to nine classes, using the disk diffusion method [25, 26]. *E*. *coli* ATCC 25922 was used as control strain. Isolates intermediate or resistant (collectively referred to as nonsusceptible) to three or more classes of antimicrobial agents were considered to be MDR [27]. In addition, isolates of the potential ESBL/AmpC collection were considered as presumptive ESBL or presumptive AmpC using the European Food Safety Authority’s criteria [28].

### Antimicrobial resistance genes

One hundred and twenty-seven (127) isolates were randomly selected (one to two isolates per sample) from the indicator *E*. *coli* collection. In addition, one isolate per virulence profile was randomly selected in each sample, resulting in 74 selected potential ExPEC isolates. These indicator *E*. *coli* and potential ExPEC isolates were examined for 14 genes encoding for resistance against streptomycin (*aadA1*), tetracycline (*tetA*, *tetB* and *tetC*), trimethoprim-sulfamethoxazole (*dfrA1*, *dfrA5* and *dfrA7*), fluoroquinolones (*qnrB*) and β-lactams (*bla*_*TEM*_, *bla*_*SHV*_, *bla*_*OXA-1*_, *bla*_*CTX-M*_ and *bla*_*CMY-2*_). In the potential ESBL/AmpC collection, 50 isolates nonsusceptible to at least one cephalosporin and originating from 15 farms, were randomly selected among 75 3GC-nonsusceptible isolates found in this study and tested for the presence of beta-lactamase genes, as previously described [21].

In addition, 14 CTX-M-positive isolates originating from six farms were tested for the CTX-M-1, -2, -8 and -9 groups by PCR. PCR amplicons were purified using QIAquick PCR purification kit (Qiagen, Germany), then forward and reverse strands of each gene were sequenced in a 3500 Genetic Analyser (Applied Biosystems) using the same PCR primer sets. Sequences were compared to known sequences using the resistance determinants database (http://www.fibim.unisi.it/REDDB/BlastInterface.asp). Primers used for screening and identification of CTX-M subtypes are available in S1 Table.

### Detection of mutations in the quinolone-resistance determining region (QRDR)

The regions of *gyrA* and *parC* genes analogous to the QRDR were amplified by PCR as described previously [29] in 76 isolates selected among 118 ciprofloxacin-resistant isolates found in the three collections and originating from 20 farms. PCR amplicons were purified as described above, and the forward strand of each gene was sequenced in a 3500 Genetic Analyser (Applied Biosystems) using the same PCR primer sets. Sequences of *gyrA* and *parC* genes were compared for each isolate with the respective reference sequences (with *E*. *coli* K-12 used as reference) using the Molecular Evolutionary Genetics Analysis software (www.megasoftware.net).

### Virulence genes and phylogenetic groups

All isolates from the indicator *E*. *coli* and potential ExPEC collections tested above for AMR genes were also examined for the presence of human ExPEC and APEC virulence genes and other virulence-associated genes that may be used to further discriminate APEC isolates as previously described [21]. In addition, some isolates from the potential ESBL/AmpC collection, carrying ESBL/AmpC genes, were tested for the same virulence genes. Each isolate was also examined by PCR to be assigned to one of the four main phylogenetic groups A, B1, B2 and D [30]. In addition, isolates belonging to phylogroups B2 or D, producers of ESBL/AmpC and/or classified potential human ExPEC, were tested by the revised phylotyping method [31].

All primers used in the PCRs performed in this study can be found in the supplementary data of our previous study [21].

### Serotyping

Thirty-nine of the 42 *bla*_*CTX-M*_- or *bla*_*CMY-2*_- positive isolates detected in this study were tested by standard agglutination methods for 86 O-serogroups described at www.ecl-lab.com/en/products/serotyping.asp [32].

### Pulsed-field gel electrophoresis (PFGE)

The 39 *bla*_*CTX-M*_*/bla*_*CMY-2*_-positive isolates were sub-typed by PFGE using *Xba*I-restriction enzyme [33]. The similarities of fragments were compared using a Dice coefficient at 1% tolerance and 0.5% optimization, and a dendrogram was generated in BioNumerics (Applied Maths) software (v. 6.6) using the UPGMA clustering method. Clusters were defined as isolates sharing at least 60% of similarity (cut-off value) as estimated by BioNumerics from the dendogram and PFGE groups as isolates sharing at least 80% of similarity.

### Plasmid characterization

Plasmid PCR-based replicon typing (PBRT) was performed on the 39 *bla*_*CTX-M*_*/bla*_*CMY-2*_-positive selected isolates for identification of 21 replicon plasmids as previously described [34, 35]. Plasmids were purified in wild type isolates using the QiaFilter Midi kit (Qiagen Inc.), following the manufacturer’s instructions. Purified plasmid DNA of 30 ESBL/AmpC-producer isolates randomly selected (16 *bla*_*CMY-2*_-positive and 14 *bla*_*CTX-M*_-positive isolates, some of which also carried the *bla*_*TEM*_ gene) was electroporated into *E*. *coli* DH10B Electromax competent cells (Invitrogen, Calsbad, CA).

Transformants were selected on Mueller Hinton agar supplemented with ceftriaxone 2 μg/ml [36]. Up to five transformants, when available, were screened by PCR for the presence of incompatibility plasmids and of all AMR and virulence genes present in the corresponding wild type strains. Transformants carrying ESBL/AmpC genes were subsequently tested for their susceptibility to the 14 antimicrobials as mentioned above.

The methodological approach that summarizes the main steps of this study can be found in S1 Fig.

### Ethics statement

The faecal samples derived from the birds were all taken from broiler production farms identified as 1 to 32. The farms were named in this way to respect their privacy. All faecal samples were collected from the floor. All other samples were taken from drinking water troughs, carcasses of birds slaughtered on farm by the owner of the farm, or water used to rinse the carcasses. Neither animal experiment nor handling of animals was conducted by the researchers. Such sampling is considered a normal diagnostic sampling method with a no impact on animal welfare. Therefore, approval by the ethics committee on the use of animals at the Cheikh Anta Diop University in Dakar, on which the École Inter-États des Sciences et Médecine Vétérinaires de Dakar depends, was not obtained.

### Statistical analysis

Prevalence of AMR by antimicrobials was estimated at the isolate and farm levels. At the isolate level, prevalence estimates were adjusted for clustering by farms and presented by type of samples. Prevalence with 95% confidence intervals of potential virulent APEC, potential ExPEC, ESBL/AmpC genes in sub-collection isolates was also estimated at the isolate and farm levels. At the isolate level, estimates were adjusted for clustering by farms and sampling weights when applicable; the weights were computed according to the probability of isolate selection in each sample. For the estimation of any prevalence at the farm level, only the faeces and drinking water samples collected on farms were considered whereas all types of samples were taken into account for estimation of prevalence at isolate level. For estimation at farm level, a farm was considered positive when at least one isolate was detected for the studied characteristics (i.e. nonsusceptible to antimicrobials, defined as MDR, potential virulent APEC or positive for AMR gene, virulence gene, QRDR mutation, etc). Prevalence estimates were carried out using the freq or surveyfreq procedure of SAS software version 9.4. To evaluate if potential risk factors from the questionnaire were associated with the presence of 3GC-nonsusceptible *E*. *coli* on farms, a chicken farm with a 3GC-nonsusceptible isolate from faeces or drinking water was defined as a case. Univariable exact logistic regressions were first performed. Potential risk factors with a p-value<0.20 from univariate models were included in a full multivariable logistic regression model. A backward selection was used to select the final model, using a p>0.05 as rejection criteria. Odds ratios (OR) with 95% interval confidence (95%IC) were used to present results. Logistic regressions were performed using the logistic procedure of SAS software version 9.4.

## Results

### Sampling and isolate collection

A total of 32 of the 34 selected farms were sampled, as the chickens on two farms had already been slaughtered a few days before our visit. A total of 50 chicken houses were sampled. One fresh fecal sample and one drinking water sample were collected in each chicken house. In addition, 10 rinsing water samples and 8 carcass swabs were obtained on farms with slaughter areas and in which slaughter operations took place at the time of our passage.

Two isolates were obtained from each sample that demonstrated growth on MacConkey (S1 Fig), except for one faecal sample and two drinking water samples from which only one lactose positive, *uidA* positive isolate was obtained, giving a total of 193 indicator *E*. *coli* isolates. In the potential ExPEC collection, a total of 90 potential ExPEC isolates positive for at least one of the targeted virulence genes were obtained in 53 samples (one to three isolates per sample) from 27 farms. Finally, in the potential ESBL/AmpC collection, up to five isolates per sample, when available, were selected resulting in a total of 130 isolates from 37 samples and 19 farms.

### *E*. *coli* isolated from healthy chickens in Senegal displayed a high prevalence of AMR

AMR was observed in the indicator *E*. *coli* isolates for all antimicrobials, the highest prevalence of nonsusceptibility being observed for tetracycline at both the isolate (92.2%) and farm (100%) levels (Table 1). For other antimicrobials, at the isolate level, the highest prevalence of nonsusceptibility was observed for sulfisoxazole (80.8%), trimethoprim-sulfamethoxazole (76.7%), streptomycin (47.7%) and nalidixic acid (44.0%), the lowest prevalence being observed for gentamicin (3.1%), amoxicillin and cefoxitin (5.7% each) and 3GC (ceftriaxone and ceftiofur, 7.2% each). At the farm level, similar trends in prevalence of nonsusceptibility were observed as for the isolate level (Table 1).

**Table 1:** Prevalence at the isolate and farm levels of antimicrobial nonsusceptibility in indicator *Escherichia coli* from healthy chickens in the region of Dakar, Senegal.

Unit of study (No. examined)	Percentage (%) of units with one or more nonsusceptible isolates per category^a^, antimicrobial class^b^ and antimicrobial^c^													
	Critically important									Highly important				
	Highest priority				High priority									
	FLQ		CPS		PEN	PEN/I	AMG			CPM	FOL		PHE	TET
	NAL	CIP	TIO	CRO	AMP	AMC	GEN	KAN	STR	FOX	SXT	SSS	CHL	TET
**Isolates**														
Drinking water/Faeces (n = 169)	44.9	27.8	8.2	8.2	37.3	6.5	3.5	13.0	49.7	6.5	77.5	81.1	15.4	91.7
Carcasses/rinsing water (n = 24)	37.5	20.8	0.0	0.0	45.8	0.0	0.0	4.2	33.3	0.0	70.8	79.2	37.5	95.8
All sources (n = 193)	44.0	26.9	7.2	7.2	38.3	5.7	3.1	11.9	47.7	5.7	76.7	80.8	18.1	92.2
**Farms (n = 32)**	84.4	59.4	28.1	28.1	68.7	21.8	12.5	34.4	87.5	21.8	93.7	96.8	43.7	100

^a^Category of human antimicrobial importance according to the World Health Organization (WHO) [37].

^b^Antimicrobial classes: (FLQ) Fluoroquinolones; (PEN/I) Penicillin+β-Lactamase inhibitors; (CPS) Cephalosporins; (PEN) Penicillin; (CPM) Cephamycin; (AMG) Aminoglycosides; (FOL) Folate inhibitors; (PHE) Phenicols; (TET) Tetracyclines.

^c^Antimicrobials: (NAL) Nalidixic acid; (CIP) Ciprofloxacin; (AMC) Amoxicillin/clavulanic acid; (TIO) Ceftiofur; (CRO) Ceftriaxone; (AMP) Ampicillin; (FOX) Cefoxitin; (GEN) Gentamicin; (KAN) Kanamycin; (STR) Streptomycin; (SXT) Trimethoprim-sulphamethoxazole; (SSS) Sulfisoxazole; (CHL) Chloramphenicol; (TET) Tetracycline.

**Fig 1:**
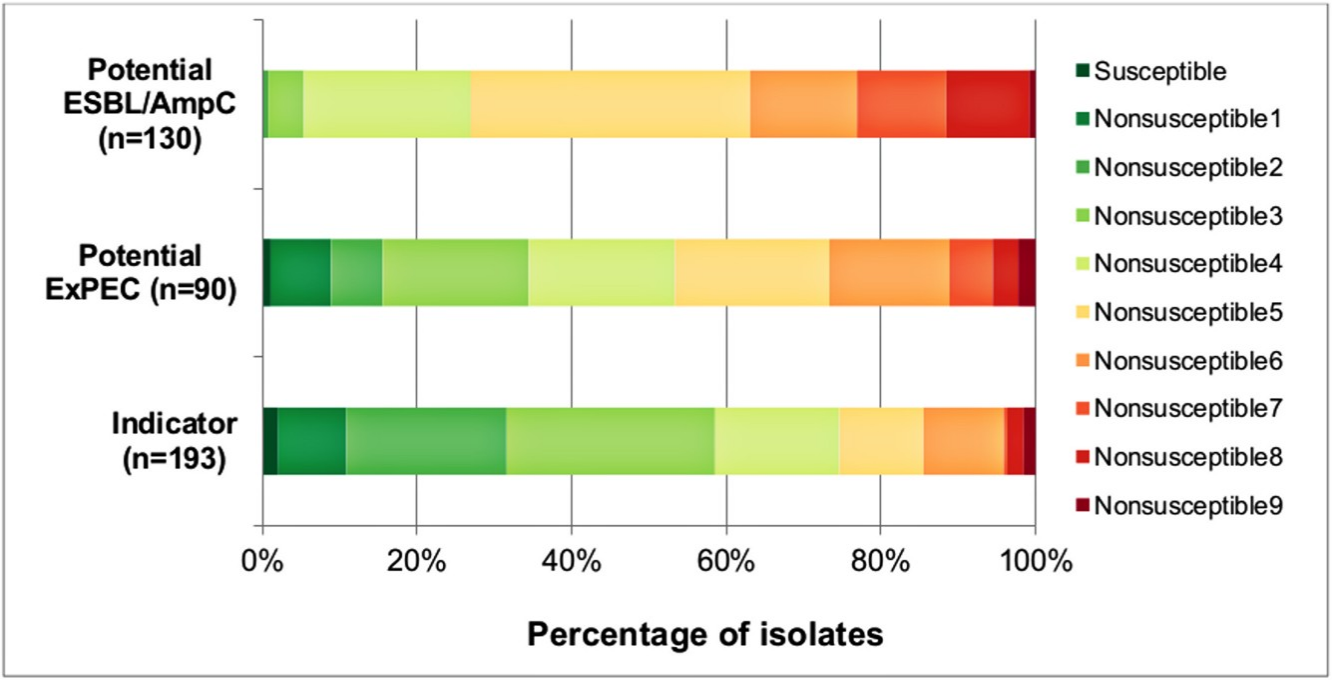
Distribution (%) of indicator (n = 193) potential ExPEC (n = 90) and potential ESBL/AmpC (n = 130) isolates from healthy chickens in the region of Dakar, Senegal according to nonsusceptibility profiles. Susceptible: susceptible to all classes of antimicrobials; Nonsusceptible 1 to 9: nonsusceptible to 1 up to 9 classes of antimicrobials; isolates nonsusceptible to 3 up to 7 antimicrobials were considered to be multidrug resistant (MDR), isolates nonsusceptible to 8 or 9 antimicrobials were considered to be possibly extensively drug resistant (XDR).

The prevalence of multidrug resistance (MDR, isolates nonsusceptible to at least one antimicrobial in at least three classes of antimicrobials) in the indicator *E*. *coli* isolates was 68.4%, with 21.2% of isolates being MDR5 or MDR6 and 3.6% of isolates classified as possible XDR (nonsusceptible to 8 or 9 antimicrobial classes, *i*.*e* extensively drug resistant isolates that remain susceptible to a maximum of 2 categories) (Fig 1). In all, 31 (96.9%; 95%CI = 83.8–99.9) farms out of the 32 investigated harbored at least one MDR indicator isolate.

Following ceftriaxone enrichment, presumptive ESBL/AmpC-producer isolates [78 isolates (60.0%; 95%CI = 36.1–80.9)], as defined by the EFSA [28], were found in 17 (53.1%; 95%CI = 34.7–70.9) of the 32 studied farms (Table 2). In contrast to the findings for the indicator *E*. *coli* collection in which isolates originating from carcasses were all 3GC-susceptible (Table 1), presumptive ESBL/AmpC isolates were observed in carcasses and carcass rinse water samples following ceftriaxone enrichment (Table 2).

**Table 2:** Prevalence at the isolate and farm levels of presumptive ESBL/AmpC-producer *Escherichia coli* isolated from healthy chickens in the region of Dakar, Senegal following enrichment with ceftriaxone.

Unit of study	No. examined	No. (%; 95%CI) of units with positive culture	No. (%; 95%CI) of units carrying presumptive ESBL/AmpC-producer isolates
**Isolates**			
Faeces and drinking water	123	N/A	75 (60.9; 95%CI = 38.4–83.5)
Carcasses and rinsing water	7	N/A	3 (42.8; 95%CI = 0.0–100)
All sources	130	N/A	78 (60.0; 95%CI = 36.1–80.9)
**Farms**	32	19 (59.4; 95%CI = 40.6–76.3)	17 (53.1; 95%CI = 35.8–70.4)

Presumptive ESBL: isolate resistant to ceftriaxone and/or ceftiofur and susceptible to amoxicillin clavulanic acid and cefoxitine; Presumptive AmpC: isolate resistant to ceftriaxone and/or ceftiofur in addition to being resistant to amoxicillin clavulanic acid and cefoxitine; Presumptive ESBL/AmpC-producers: cumulative of presumptive ESBL-producers and presumptive AmpC-producers; N/A: Not applicable.

Notably, in the potential ESBL/AmpC a lower proportion of isolates nonsusceptible to most antimicrobials, except for ceftriaxone and ceftiofur, for which 58.5% of isolates were nonsusceptible, was observed (S2 Table). In addition, almost all potential ESBL/AmpC isolates were classified as MDR; 11.5% of potential ESBL/AmpC isolates were classified as MDR7, and 11.5% were defined as possible XDR (Fig 1).

### Prevalence of AMR genes

One or more AMR genes were detected in 111 (87.4%) of the 127 tested indicator *E*. *coli* isolates. The most prevalent AMR genes were those encoding resistance against tetracycline (*tet* genes) and trimethoprim-sulfamethoxazole (*dfrA* genes) (Table 3). Genes encoding resistance against streptomycin [*aadA1* (19.7 (10.9–28.4)] or ciprofloxacine [*qnrB* (11.0%; 95%CI = 5.4–16.6)] were also detected and among ESBL/AmpC genes, *bla*_*TEM*_ (36.2%; 95%CI = 26.3–46.1) was the most frequently detected β-lactamase gene. Most of the AMR genes were found in the four types of samples with the exception of *dfrA5* which was only found in isolates from drinking water and faeces, *bla*_*CTX-M*_ which was only found in isolates from faeces and *bla*_*SHV*_, *bla*_*OXA-1*_ and *bla*_*CMY-2*_ which were only found in isolates from drinking water (S3 Table). At the farm level, the most prevalent AMR genes were *tetA* [27 isolates (84.4%; 95%CI = 67.2–96.9)], *dfrA7* [25 isolates (78.1; 60.0–90.9)], *bla*_*TEM*_ [19 isolates (59.4; 95%CI = 40.6–76.3) each] and *dfrA1* [18 isolates (56.3; 95%CI = 37.7–73.6)].

**Table 3:** Prevalence at the isolate and farm levels of AMR genes in 127 indicator *Escherichia coli* isolated from healthy chickens in the region of Dakar, Senegal.

AMR gene	Isolate level (n = 127)		Farm level (n = 32)	
	No. of positive isolates	Prevalence (95% CI)	No. of positive farms	Prevalence (95% CI)
*tetA*	73	57.5 (47.8–67.2)	27	84.4 (67.2–96.9)
*tetB*	25	19.7 (12.6–26.8)	14	43.7 (26.4–62.3)
*dfrA1*	37	29.1 (17.6–40.7)	18	56.3 (37.7–73.6)
*dfrA5*	13	10.2 (3.6–16.9)	9	28.1 (13.7–46.7)
*dfrA7*	61	48.0 (35.7–60.3)	25	78.1 (60.0–90.9)
*qnrB*	14	11.0 (5.4–16.6)	10	31.3 (16.1–50.0)
*aadA1*	25	19.7 (10.9–28.4)	11	34.4 (18.6–53.2)
*bla*_*TEM*_	46	36.2 (26.3–46.1)	19	59.4 (40.6–76.3)
*bla*_*OXA-1*_	1	0.8 (0.0–2.3)	1	3.1 (0.0–9.2)
*bla*_*SHV*_	2	1.6 (0.0–3.7)	2	6.3 (0.8–20.8)
*bla*_*CTX-M*_	2	1.6 (0.0–4.8)	1	3.1 (0.0–9.2)
*bla*_*CMY-2*_	1	0.8 (0.0–2.4)	1	3.1 (0.0–9.2)

AMR, antimicrobial resistance; No., Number; prevalence expressed in percentage; 95%CI, 95% confidence interval. Tetracycline resistance gene *tetC* was not detected in any isolate.

A total of 251 isolates (including all collection isolates) from 32 farms were tested for ESBL/AmpC genes. The *bla*_*CTX-M*_ genes were detected in 14 isolates (5.6%, 95% CI = 1.4–9.8) and *bla*_*CMY-2*_ in 28 isolates (11.2%, 95% CI = 2.7–19.6). The *bla*_*CTX-M*_ gene was detected in six farms and *bla*_*CMY-2*_ in seven farms; both genes were simultaneously present in three of these farms. In addition, *bla*_*CTX-M*_ positive isolates were obtained from 13 of the 141 faecal isolates and 1 of the 16 rinsing water isolates, whereas *bla*_*CMY-2*_ was detected in 6 of the 75 isolates obtained from drinking water samples and 22 faecal isolates. The 14 *bla*_*CTX-M*_ -isolates detected in this study were of genotypes *bla*_*CTX-M-1*_ (9 isolates found in 4 farms), *bla*_*CTX-M-8*_ (3 isolates found in 2 farms), and *bla*_*CTX-M-15*_ (2 isolates, each from a separate farm). The *bla*_*CTX-M*_ identified in rinsing water was of genotype *bla*_*CTX-M-1*_. In addition, the *bla*_*CTX-M-15*_ positive isolates belonged to phylogenetic groups A and D for which the O serogroups were non-typable and those of *bla*_*CTX-M-8*_ genotype were of phylogenetic groups B1 (2 isolates) and A (1 isolate); these isolates were also non-typable. The *bla*_*CTX-M-1*_ positive isolates belonged to phylogroups A (6 isolates of which 5 were of serogroup O45), D (2 isolates) and B1 (1 isolates).

### Mutations in the quinolone-resistance determining region (QRDR) of *gyrA* and *parC* genes

Seventy-six of the 118 ciprofloxacin-resistant isolates found in this study, originating from 20 farms and selected from the three collections, were sequenced for the QRDR of *gyrA* and *parC* genes. Two mutation positions were observed in each QRDR gene and 74 (97.4%; 95%CI = 93.2–100) isolates carried one or more mutations in *gyrA* or *parC* (Table 4). In *gyrA*, the most frequent mutations were the substitution of aspartic acid (Asp) by asparagine (Asn) at position 87 (69 isolates) and the substitution of serine (Ser) by leucine (Leu) at position 83 (71 isolates). In *parC* gene, the most frequently observed mutation was the substitution of serine by isoleucine (Ile) at position 80 (69 isolates). Sixty-two isolates (82.2%; 95%CI = 76.5–87.9) demonstrated a combination of the 3 same mutations, Ser83Leu and Asp87Asn in *gyrA* and Ser80Ile in *parC* and these isolates originated from 19 (95.0%; 95%CI = 75.1–99.8) of the 20 farms. In addition, 67 (88.2%; 95%CI = 81.8–94.5) isolates were carriers of double-serine mutation (*gyrA* Ser83Leu and *parC* Ser80Ile).

**Table 4:** Presence of mutations in the *gyrA* and *parC* genes in 76 ciprofloxacin-resistant *Escherichia coli* isolated from 20 healthy chicken farms in the region of Dakar, Senegal.

Amino acid changes in *gyrA*		Amino acid changes in *parC*		No. of isolates carrying mutation(s) (% and 95%CI)	No. of farms of origin (% and 95%CI)
Ser83	Asp87	Ser80	Glu84		
Leu	Asn	Ile	-	62 (82.2; 76.5–87.9)	19 (95.0; 75.1–99.9)
Leu	Asn	-	Lys	1 (1.7; 0.0–5.3)	1 (5.0; 0.1–24.8)
Leu	Asn	Ile	Gly	2 (2.3; 0.0–5.6)	2 (10.0; 1.2–31.7)
Leu	Asn	Arg	-	1 (1.9; 0.0–5.2)	0 (0.0; 0.0–16.8) ^a^
Leu	Asn	-	-	2 (2.3; 0.0–5.7)	2 (10.0; 1.2–31.7)
Tyr	Asn	-	-	1 (0.8; 0.0–2.2)	1 (5.0; 0.1–24.8)
Leu	-	Ile	-	3 (4.7; 0.0–9.5)	2 (10.0; 1.2–31.7)
-	-	Ile	-	2 (1.9; 0.0–5.9)	2 (10.0; 0.0–23.2)
-	-	-	-	2 (1.7; 0.0–4.5)	2 (10.0; 1.2–31.7)

^a^Prevalence at farm level was computed using feaces and drinking water sample and the isolate carrying these mutations was from rinsing water; Ser, serine; Leu, leucine; Tyr, tyrosine; Asp, aspartic acid; Asn, asparagine; Ile, isoleucine; Arg, arginine; Glu, glutamic acid; Lys, lysine.

### Potential human ExPEC are found in *Escherichia coli* isolated from healthy chickens in Senegal

The 127 indicator *E*. *coli* isolates were distributed among the four phylogroups A (n = 59), B1 (n = 56), B2 (n = 5) and D (n = 7). Overall, 73 (57.5%) of indicator *E*. *coli* isolates carried at least one of the virulence genes under investigation. All virulence genes associated with APEC were detected and among genes associated with human ExPEC, *iucD*, *kpsMII* and *papC* were observed. The virulence genes *cnf*, *sfa/foc*, and *afa/dra* were not detected in any isolate. Moreover, 32 combinations of virulence genes were observed. Of these combinations, *ompT* (n = 13 isolates), *iroN/iss/hlyF/ompT* (n = 7 isolates), *iroN/iss/hlyF*, *iroN/iss/hlyF/ompT/iucD* and *iroN/iss/ompT/iucD/tsh* (n = 6 isolates for each) were most frequently observed. Each combination originated from at least two farms and among the four phylogenetic groups. The five virulence genes associated with APEC (*iroN*, *iss*, *hlyF*, *ompT* and *iucD*) and the *tsh* gene were detected in 80% to 100% of isolates of phylogroup B2 and 71% of phylogroup D-isolates were carriers of virulence genes *iss*, *hlyF* and *ompT*.

**Fig 2:**
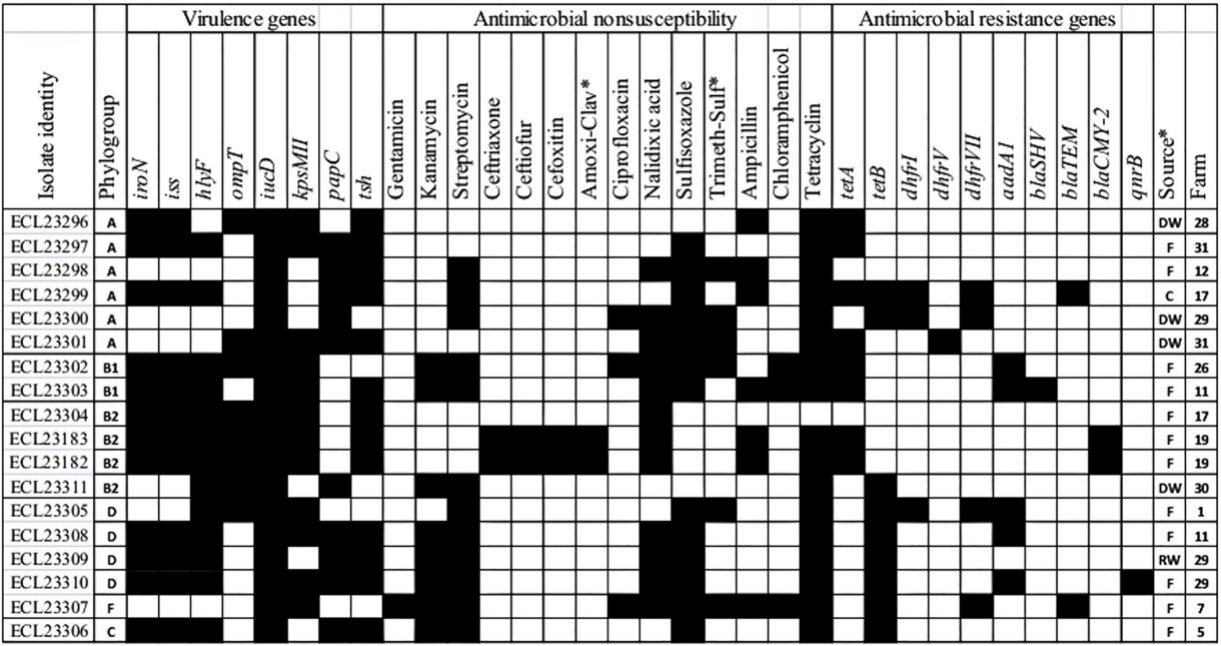
Virulence and antimicrobial resistance characteristics of isolates defined as potential human ExPEC based on their virulence profiles. (Amoxi-clav) amoxicillin/clavulanic acid; (Trimeth-sulf) trimethoprim-sulfamethoxazole. Black boxes indicate antimicrobial nonsusceptibility, the presence of virulence genes or of AMR genes. No isolate was positive for any of the *bla*_*CTX-M*_, *bla*_*OXA-1*_, *tetC*, *afa/dra*, *sfa/foc* or *cnf* genes, hence these genes were removed. Sources of isolates: (F) faeces; (DW) drinking water; (C) carcass; (RW) rinsing water.

Based on the presence of four or five of the five APEC-associated virulence genes, 22 (17.3%; 95%CI = 7.3–27.3) of the 127 indicator *E*. *coli* isolates and 9 farms (28.1%; 95%CI = 13.8–46.8), were defined as virulent APEC, of which four were also classified as potential human ExPEC. The ExPEC sub-collection of 74 isolates permitted the detection of an additional 43 (55.6%; 95%CI = 42.8–68.2) potential virulent APEC and 13 (15.6%; 95%CI = 5.7–25.4) potential human ExPEC isolates. In addition, one isolate from the potential ESBL/AmpC collection was also defined as a potential human ExPEC isolate. This results in a total of 18 potential human ExPEC isolates belonging to phylogroups A (6 isolates), B1 (2 isolates), B2 (4 isolates of which two were *bla*_*CMY-2*_-positive), D (4 isolates), F (1 isolate) and C (1 isolate) from 12 farms (Fig 2). In addition to being defined as potential human ExPEC, these isolates were mostly carriers of at least three of the genes defining APECs. In addition, almost all of these potential human ExPEC were MDR, some of which were resistant to critically important antimicrobials in human health (3GC, gentamicin and ciprofloxacin). Moreover, almost all potential human ExPEC were positive for at least one of the AMR genes investigated.

Examination of the 10 potential human ExPEC belonging to phylogroups B2 (n = 3) et D (n = 7) using the revisited phylotyping method [31] showed all B2-isolates remained in the same phylogroup, whereas among the seven D-isolates, 4 remained D-isolates and the others were reassigned to phylogroup B2, C and F (1 isolate, each) (Fig 2). For the 16 other isolates (all ESBL/AmpC-producers), the two B2-isolates still belonged to group B2 and the 14 D-isolates were reassigned to phylogroups E (11 isolates), B2 (2 isolates) and F (1 isolate).

### Some ESBL/AmpC-producing *E*. *coli* from healthy chickens of different farms may be clonally related

**Fig 3:**
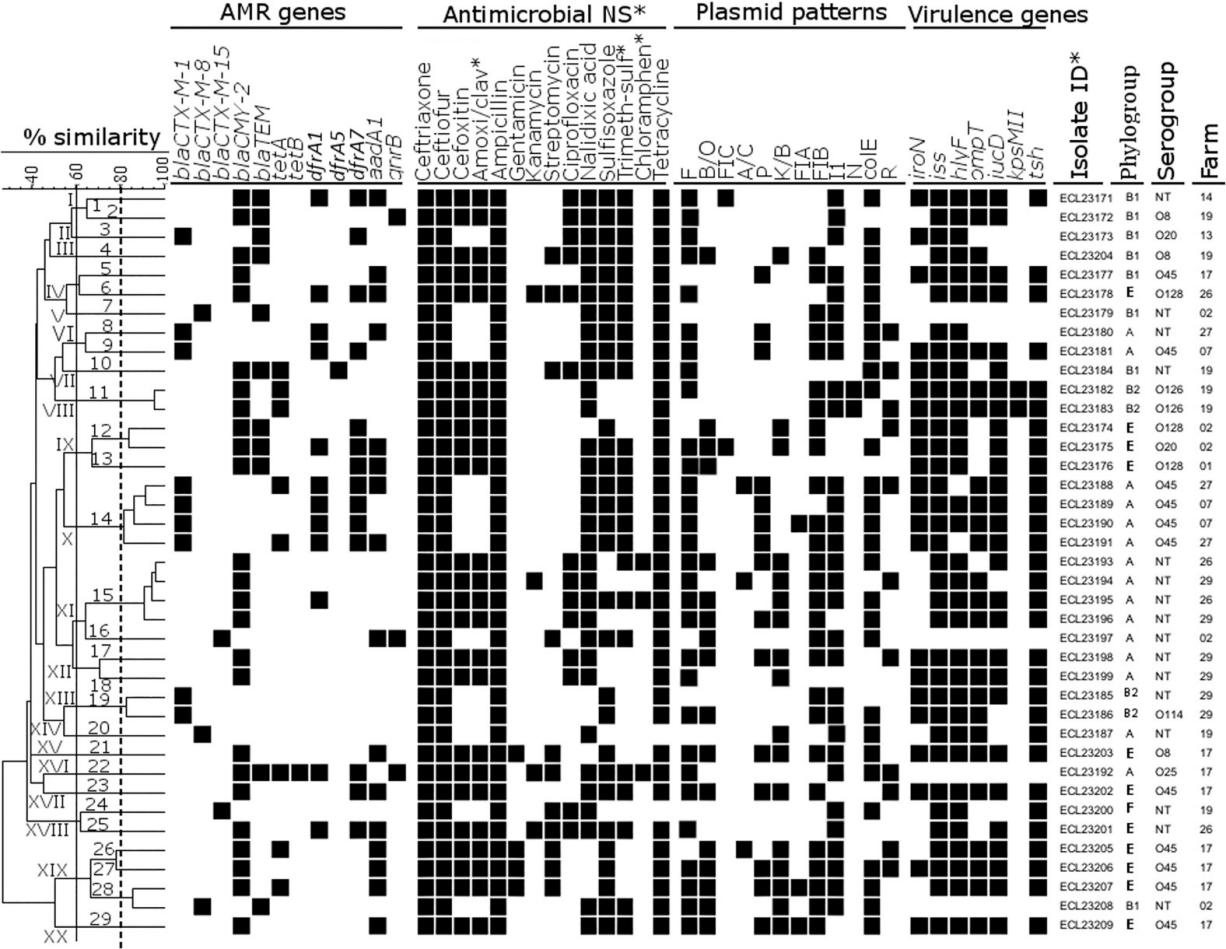
Clustering analysis of genetic variation of 39 *bla*_*CTX-M*_/*bla*_*CMY-2*_-positive *Escherichia coli* isolates from healthy chickens in the region of Dakar, Senegal. The dendrogram was generated using Dice coefficient and the unweighted pair-group method and arithmetic average (UPGMA). Based on a similarity index of ≥ 60% (continuous line), 23 major clusters (I-XXIII) were found inside which 32 PFGE groups (in arabic numerals) were identified when the similarity was set at 80% (broken line). (AMR) antimicrobial resistance; (antimicrobial NS), antimicrobial nonsusceptibility; (Amoxi/clav), amoxicillin-clavulanic acid; (Trimeth-sulf), trimethoprim-sulfamethoxazole; (Chloramphen), chloramphenicol; (Isolate ID), isolate identity. None of the ESBL/AmpC-producing isolates were positive for AMR genes *bla*_*OXA-1*_ or *tetC* and as none were carriers of virulence genes *papC*, *sfa/foc*, *afa/dra* or *cnf* these genes were removed from the dendrogram.

PFGE showed a high genetic diversity of ESBL/AmpC-producing *E*. *coli* isolates (Fig 3). Based on a similarity threshold set at 60%, 20 clusters (I-XX) were observed, of which 11 consisted of at least two isolates (Fig 3). Within these clusters, 29 PFGE groups (1–29) were found when the similarity between isolates was set at 80%. Among these, six (PFGE groups 11, 12, 14, 15, 19 and 28) consisted of at least two isolates and the remaining 23 were singletons. There were two major PFGE groups: group 14 (within cluster X) includes four *bla*_*CTX-M-1*_-isolates belonging to serogroup O45 and to phylogenetic group A, and group 15 (within cluster XI) includes four *bla*_*CMY-2*_-isolates belonging to phylogroup A for which the serogroup was untypeable in our system. Isolates of PFGE group 14 were from 2 distinct farms (farms 07 and 27) as were those of PFGE group 15 (farms 26 and 29) and PFGE group 28 (farms 02 and 17) (Fig 3). In addition, isolates of PFGE group 14 were all nonsusceptible to 3GC, nalidixic acid, sulfisoxazole, ampicillin, tetracycline and trimethoprim-sulfamethoxazole, and all possessed AMR genes *bla*_*CTX-M-1*_, *dfrA1* and *dfrA7*. Isolates of PFGE group 15 were nonsusceptible to all beta-lactams, nalidixic acid, ciprofloxacin and tetracycline. Most isolates of both PFGE groups 14 and 15 carried at least four virulence genes and many were defined as potential highly virulent APEC. Furthermore, the two isolates of PFGE group 11 that belonged to phylogroup B2 and produced *bla*_*CMY-2*_ were multi-drug resistant and defined as potential human ExPEC.

### ESBL/AmpC-producing *E*. *coli* from healthy chickens often possess several plasmid replicon types

Plasmid replicon typing identified 12 plasmid types (Fig 3). Each isolate carried at least two plasmids, with a maximum of eight. The most prevalent plasmid incompatibility groups were F (76.9%), colE (73.7%), FIB and I1 (69.2% each). In addition, plasmids F, I1 and colE were detected in samples of faeces, drinking water and rinsing water; FIA, FIC and N in faeces only; the others (A/C, B/O, K/B, P, FIB and R) in samples of faeces and drinking water (Fig 3). Furthermore, most of the *bla*_*CTX-M-1*_ positive isolates harboured plasmids F, P, FIB, I1 and colE, those positive for genotype *bla*_*CTX-M-8*_ were associated with the plasmids FIB, I1 or colE and the isolates positive for *bla*_*CTX-M-15*_ were carriers of I1 or colE (Fig 3).

### Plasmids carrying *bla*_*CTX-M*_ or *bla*_*CMY-2*_ in ESBL/AmpC-producing *E*. *coli* from healthy chickens may be transferred and often carry resistance to other non beta–lactam antimicrobials

**Fig 4:**
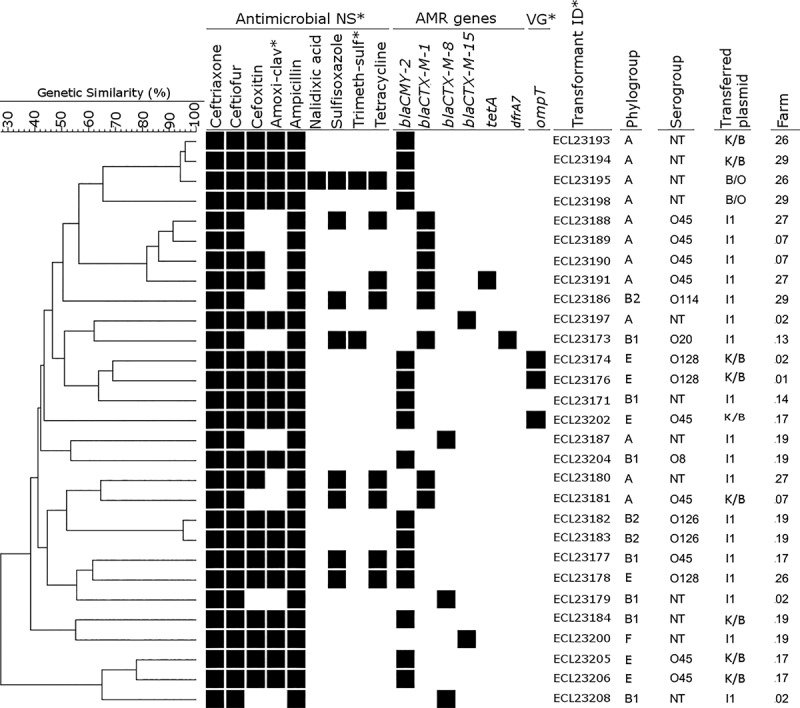
Dendrogram of 29 CTX-M or CMY-2 transformants, showing antimicrobial nonsusceptibility, antimicrobial resistance genes, and virulence genes co-transferred with ESBL/AmpC genes and replicon plasmids carrying these genes. (Antimicrobial NS) Antimicrobial nonsusceptibility; (Amoxi-clav) amoxicillin-clavulanic acid; (Trimeth-sulf), Trimethoprim-sulfamethoxazole, (VG), Virulence gene; (Transformant ID) Transformant identity.

Plasmids carrying ESBL/AmpC genes were successfully transferred by electroporation for 29 of the 30 examined isolates. The *bla*_*CMY-2*_ gene was carried on replicon plasmids K (n = 8), I1 (n = 6) or B/O (n = 2) and *bla*_*CTX-M*_ genes were located on I1 (n = 12) or K (n = 1). I1 carriers of the *bla*_*CMY-2*_ gene were detected on four farms and those with *bla*_*CTX-M*_ on seven farms; the simultaneous presence of both types of I1 was identified on two farms. *bla*_*CMY-2*_-bearing K plasmids were present on six farms whereas the *bla*_*CTX-M*_-bearing K plasmid was identified on a separate farm. The B/O carrying *bla*_*CMY-2*_ was detected in two farms in which both types of I1 and *bla*_*CMY-2*_-bearing K were identified (Fig 4). Some plasmids carrying ESBL/AmpC genes, such as B/O, I1, and K, also transferred resistance to other antimicrobials such as nalidixic acid, tetracycline, sulfisoxazole and trimethoprim-sulfamethoxazole, sometimes with corresponding tested AMR genes. In addition, three *bla*_*CMY-2*_-bearing K plasmids co-transferred the *ompT* virulence gene.

### Antimicrobial use and risk factors associated with presence of cephalosporin-nonsusceptible isolates on farms

Of the 32 farms studied, five had used an anticoccidial drug for preventive purposes in the studied batch, two had used a quinolone (enrofloxacin or norfloxacin) for curative purposes, and one had used tetracycline both to prevent and cure disease; the remaining 24 farms did not report any drug use. Two of the five farmers who used the anticoccidials and the one who used tetracycline said they did not have veterinary training and did not use the services of any veterinarian or person with skills in the field. Among the potential risk factors, trained staff, farms with at least two buildings housing chickens, the lack of observance of an empty period, allowing visitors on farms, and disposing of dead chickens into the environment were selected for inclusion (p<0.20 in univariable analysis) in a multivariate logistic regression modelling the presence of 3GC-nonsusceptible indicator *E*. *coli* isolates on farms (Table 5). However, none was found to be significantly associated with presence of 3GC-nonsusceptible indicator *E*. *coli* isolates on farms in the reduced model.

**Table 5:** Potential risk factors associated with third-generation cephalosporin-resistant *Escherichia coli* isolates on chicken farms in the region of Dakar, Senegal.

Potential risk factor collected on batch under study	No. of farms	% of farms with 3GC resistant isolates detected	Odds ratio (univariable analyses)		
			Estimate	95% CI	p-value
Therapeutic use of antimicrobials					
Yes	8	25.0	0.8	0.13–5.0	0.82
No	24	29.2	Ref.		
Trained staff : staff with veterinary skills					
Yes	5	60.0	5.2	0.7–39.0	0.10*
No	27	22.2	Ref.		
Number of chicken houses (buildings on the farm in which the chickens are raised)					
≥ 2	18	38.9	3.8	0.6–22.4	0.13*
1	14	14.3	Ref.		
Presence of health problem in the cohort according to the farmer					
Yes	12	33.3	1.5	0.3–7.2	0.61
No	20	25.0	Ref.		
Use of growth promoters at least once in the cohort (according to the farmer)					
Yes	17	35.3	2.2	0.4–10.9	0.34
No	15	20.0	Ref.		
Cohabitation (chicken house is within human habitation or is shared with other animal species)					
Yes	12	33.3	1.5	0.3–7.2	0.61
No	20	25.0	Ref.		
Type of construction for the chicken “house”					
Clay	6	16.7	0.4	0.04–4.5	0.49
Concrete	26	30.7	Ref.		
Observance of an empty period between chicken batches					
No	3	66.7	6.3	0.5–80.2	0.15*
Yes	29	24.1	Ref.		
Visitors allowed on the farm					
Yes	29	24.1	0.1	0.01–2.0	0.15*
No	3	66.7	Ref.		
Contact of the staff with other farms					
Yes	10	40.0	2.3	0.4–11.3	0.31
No	22	22.7	Ref.		
Water source*					
Borehole or distribution network	14	28.6	1.0	0.2–4.9	0.96
Well water	18	27.8	Ref.		
Dead chicken carcass disposal					
In the environment	15	40.0	3.1	0.6–15.7	0.16*
Burial/cremation	17	17.7	Ref.		
Type of operation (yes when the farm only exploits broilers, not when it is a mixed farm)					
Mixed farm	8	33.3	0.3	0.03–2.7	0.27
Only broilers	24	12.5	Ref.		

*: Variable selected for inclusion in the multivariable model (p<0.20)

## Discussion

This study has demonstrated the presence of ESBL/AmpC-producer and potential ExPEC isolates in healthy chickens in Senegal. Given that a large proportion of the farms in the study area (32 out of 68) were randomly selected, our results are likely representative of the Dakar region of Senegal.

The observed high proportion (>68% in indicator isolates) of MDR isolates, with 96.9% of the 32 farms harboring at least one MDR indicator isolate, suggests that therapeutic options could be limited in the treatment of associated diseases. We have already reported a high (86.2%) prevalence of MDR *E*. *coli* isolates from diseased chicken farms in Senegal [21], and the prevalence of MDR we found here at the farm level is also in line with the 94% of MDR farms reported in Punjab, India [38].

A high percentage (58.5%) of 3GC-nonsusceptible isolates was observed in the potential ESBL/AmpC collection suggesting chickens and their environment in Senegal could act as reservoir of 3GC-resistant *E*. *coli*. The high prevalence of 3GC-nonsusceptible isolates is also indicative of the selection pressure that antimicrobial use in animals could generate, and underlines the importance of making a careful choice in the measures to be taken to control antimicrobial use in Senegal. In fact, it has been reported in Senegal that antimicrobials, including those of critical importance in human health, can be purchased from street vendors and used in chickens [20]. Indeed, in our field investigation, one farmer had used norfloxacin in chickens, a fluoroquinolone used in the treatment of urinary tract infections in humans. To be effective, this control must be extended to veterinary antimicrobials such as ceftiofur, for which a prevalence of nonsusceptibility similar to that of ceftriaxone was observed in all collections in the present study. Indeed, ceftiofur is a 3GC reserved for veterinary use only; nevertheless, *in ovo* use of ceftiofur has been associated with 3GC-resistant enterobacteria in human and chickens [39].

The ciprofloxacin resistance observed in this study could be due to widespread use of quinolones in chickens in Senegal to treat or prevent bacterial infections in the field. Nevertheless, therapeutic use of enrofloxacin or norfloxacin was only reported in two of the 20 farms harboring ciprofloxacin-resistant isolates in our study. Our results show the presence of *E*. *coli* with at least 3 mutations in the QRDR of *gyrA* (2 mutations) and *parC* (1 mutation) in 95% of tested farms, clearly reflecting a high level of resistance to all fluoroquinolones and suggesting a widespread use of fluoroquinolones in these farms or in the chick hatcheries, two or more *gyrA* or *parC* substitutions being needed for a high level of resistance [40]. We have already described these mutations in our previous characterisation of clinical isolates in Senegal [21]. In addition, we have found in the present study amino acid changes Ser80Tyr in *gyrA* and Ser80Arg, Glu84Lys and Glu84Gly in *parC*, which were also reported in *E*. *coli* by other studies [15, 41]. Only two of the 68 ciprofloxacin-resistant isolates without presence of *qnrB* gene did not carry mutations in *gyrA* and/or *parC* and ciprofloxacin-resistance in these two isolates could be due to the presence of other *qnr* genes or efflux pump genes not explored in this study. Sixty-seven (88.2%) isolates were carriers of the double-serine mutations in *gyrA* (Ser83Leu) and *parC* (Ser80Ile). This double mutation has been described as a fitness factor that helps the ST131 pandemic clone to successfully spread into new ecological niches [42]. Indeed, our isolates carrying this double mutation were found in 19 out of 20 farms, suggesting that they may have spread from a single clone, but further study is needed to elucidate this hypothesis.

A discrepancy was observed between the high AMR prevalence and the low reported use of antimicrobials (24 farmers did not report any drug use). It is possible that breeders used these antimicrobials but did not report their use, resulting in an information bias. Use in previous flocks is also possible, which was not asked in our questionnaire. Farms for which the staff reported having veterinary skills demonstrated a greater tendency to have 3GC-resistant isolates compared to farms for which the staff reported having no veterinary skills. This suggests that breeders with veterinary knowledge are using more antimicrobial agents. It has been reported that most farmers in Senegal have received basic training in breeding techniques and may go beyond their role by carrying out prohibited acts such as the prescription and use of antimicrobials, often being under-dosed [43]. Hence, breeders may hesitate to declare all antimicrobial use. In Senegal, antimicrobials such as quinolones, tetracyclines and sulfonamides are generally used in poultry farms as anti-stress, anti-coccidian or anti-infective drugs [44]. Therefore, the high percentage of resistance reported here for nalidixic acid, sulfonamides, and aminoglycosides may be a consequence of the widespread use of these antimicrobials in poultry. It has been reported that for some chicken farms in Senegal no validated program of prophylaxis is applied and some breeders acquire antibiotics often marketed for human medicine, by fraudulent means or through street vendors [20].

Apart from the potential under-reporting of antimicrobial use, another plausible explanation for the high prevalence of QRDR mutations and 3GC-resistance could be their use in local hatcheries, in hatching eggs imported from Europe or Latino America [20] or even in humans and other animal species living in close proximity with chickens. In addition, in Senegal, most poultry farmers in Senegal use Virkon as a disinfectant [45]. As Virkon has been shown to promote the selection of antimicrobial resistance through QRDR mutation in enterobacteria [46], the mutations could therefore be maintained without quinolone-exposure. Similarly, the use of hydrogen peroxide, a disinfectant of the same class as Virkon, has been reported to increase the resistance of avian *E*. *coli* to ceftiofur, cefoxitin and amoxicillin/clavulanic acid [16]. Therefore, the widespread use of Virkon in chicken farms in Senegal may also have contributed to 3GC resistance.

The *bla*_*CMY-2*_ (0.8%) and *bla*_*CTX-M*_ (1.6%) genes were detected in very low proportions among indicator *E*. *coli* isolates in our study whereas the proportion of isolates positive for *bla*_*TEM*_ was higher (36.2%). Prevalence of the *bla*_*CTX-M*_ and *bla*_*TEM*_ genes in indicator *E*. *coli* isolates is very different from that reported in chickens in another African country, Zambia [47], where *bla*_*CTX-M*_ was more prevalent (18.5%) than *bla*_*TEM*_ (6.0%); the authors did not examine their isolates for the presence of *bla*_*CMY-2*_. These findings may be due to a difference in antimicrobial use in Senegal compared to Zambia. The study in Zambia did not identify the antimicrobials used on the farms investigated. In addition to the use of 3GCs, the use of aminopenicillins [48], certain other antimicrobials or even feed additives such as zinc or copper [49] in chickens can lead to the selection of *E*. *coli* producers of the ESBL CTX-M. The *bla*_*CTX-M*_ genes detected in this study were of genotype *bla*_*CTX-M-1*_, _*-8*_ and _*-15*_. In our previous study on clinical isolates from chickens in Senegal [21], *bla*_*CTX-M*_ genes were also found to be of genotypes *bla*_*CTX-M-1*_ and *bla*_*CTX-M-8*_, but this is the first study reporting *bla*_*CTX-M*-15_-producing *E*. *coli* isolated from chickens in Senegal. The latter, in recent years has become the predominant *bla*_*CTX-M*_ variant in human isolates in many areas of the world [50]. The presence of isolates carrying *bla*_*CTX-M*-15_ in poultry farms in Senegal, together with the fact that *bla*_*CTX-M*-15_-producing *E*. *coli* have also been isolated from children in a remote Senegalese village [51], suggest that this variant may be well established in enterobacteria of both human and animal origin in Senegal. In addition, the *bla*_*CTX-M*-15_ genes were identified on the epidemic plasmid I1, highlighting the importance of monitoring *bla*_*CTX-M*-15_-positive *E*. *coli* at both the farm and community levels. Nevertheless, the *bla*_*CTX-M*-15_ positive isolates identified in our study possess fewer virulence genes than the *bla*_*CTX-M-1*_-producing isolates.

Genes encoding resistance to tetracyclines and trimethoprim-sulfamethoxazole were detected in a high proportion of indicator *E*. *coli* isolates, as was the presence of phenotypical resistance in these isolates. Although the present study did not demonstrate significant use of these antimicrobials, previous studies in Senegal have reported their use in chickens as growth promoters or for treatment [44] and in humans for treatment of different bacterial diseases [52, 53].

The finding of ESBL/AmpC-producing isolates in faeces, drinking water and carcass rinsing water samples underlines the risk to the human population both via carcass contamination or direct contact with chickens given the proximity of poultry and humans in Senegal. In Senegal, as in many developing countries, chicken farms can be established in areas densely populated, and birds can be sold live in markets. This danger may be exacerbated by the finding of potential human ExPEC isolates, some of which produce ESBL/AmpC, as observed in several farms. Among these potential human ExPECs, were two isolates of the serogroup O126 in the PFGE group 11, belonging to phylogroup B2, being *bla*_*CMY-2*_-producers. Indeed, pathogenic *E*. *coli* belonging to phylogroup B2 commonly cause death in humans [54]. Thus, the presence of such isolates in an environment close to humans could facilitate their transmission through direct or indirect contact. This demonstrates the importance of compliance with biosecurity measures in poultry farms in Senegal, especially since our survey showed that visitors can freely enter the farms and that dead birds may be disposed into the immediate environment.

The finding that ESBL/AmpC-producing isolates of different farms belonged to a single PFGE group suggests their potential circulation between farms. Alternatively, these clonally related ESBL/AmpC-producing isolates could have originated from a single source such as a same hatchery as we suggested in our previous study [21] and has been observed by several authors [48, 55, 56]. Another explanation for the spread of resistance genes could be the horizontal transmission of plasmids bearing these ESBL/AmpC genes. Effectively, the CTX-M genes were identified on plasmids of incompatibility groups I1 and K/B and CMY-2 gene on plasmids K/B, I1 and B/O. All isolates of the PFGE group 14 carried CTX-M-1 genes on the plasmid I1 and the two most closely related isolates of the PFGE group 15 carried the CMY-2 gene on K plasmids, indicating that these plasmids have spread with these clonally related isolates. However, I1 and K plasmids carrying ESBL/AmpC genes were also found in different clusters, suggesting the involvement of a horizontal dissemination as observed by Castellanos et *al*. [57], who reported the horizontal dissemination of ESBL/AmpC genes via plasmids found in highly heterogeneous avian *E*. *coli* strains. Nevertheless, the finding that isolates of the same PFGE group carry identical plasmids suggests that AMR is disseminated either by clones, via plasmids or by both plasmids and clones as observed by Agersø and colleagues [48]. Therefore, further experiments using whole genome sequencing are required to elucidate the mechanism of spreading of ESBL/AmpC genes. Interestingly, I1 plasmids, which were found to carry *bla*_*CTX-M*_ or *bla*_*CMY-2*_, were found in almost all clusters and on different farms that used antimicrobials for therapeutic purpose or as growth promoters. It should be noted that the I1 plasmid is considered as “epidemic” with its occurrence linked to antimicrobial use [58] and therefore its spread must be monitored as well as the AMR itself. It should also be noted that other factors such as migratory birds [59] or the practice in Senegal of using intermediaries to collect live or killed chickens on farms for sale in the markets [20] may also be an important source of pathogen spread.

This study showed that O45 (20.0%) was the most prevalent O serogroup among ESBL/AmpC-producing *E*. *coli*. These O45-isolates belonged to phylogroup A, B1 or D and were all defined as MDR and potentially virulent APEC. Mora et *al*. [60] also reported serogroup O45 belonging to phylogroups A, B1 and D among *E*. *coli* isolated from diseased chickens. These authors found that the O45:D isolates were also MDR and constituted a specific clonal group in birds in contrast to O45:B2 isolates which were found in both humans and birds. Thus, the O45 isolates in our study would likely not pose a risk to humans, but could potentially cause colibacillosis in chickens. On the other hand, none of the isolates belonged to the serogroups most frequently isolated from cases of colibacillosis in chickens, such as O1, O2 and O78 [61, 62], as we also reported in our previous study of *E*. *coli* isolated from cases of chicken colibacillosis in Senegal [21]. Nevertheless, based on the defining criteria [21], 22 (17.3%) indicator *E*. *coli* isolates were defined as potentially virulent APEC of which 4 were potentially capable of infecting humans according to previously established criteria [63]. Some APEC isolates share certain genetic similarities with human ExPEC, thus being potentially capable of infecting humans [5]. In addition, it has been shown that chicken fecal *E*. *coli* isolates possessing ExPEC-associated genes can cause ExPEC-associated illnesses in animal models for human infections [23]. These findings suggest that chickens and/or their environment in Senegal, in addition to being a reservoir of AMR, are a possible reservoir of *E*. *coli* that are potentially pathogenic for humans.

Although some potential risk factors, including management practices, had a tendency to be associated with the presence of 3GC-resistant isolates on farms, no risk factors for presence of 3GC-nonsusceptible isolates on farms were statistically significant in the multivariate model. Studies in Vietnam [64] and in countries of the South Western Indian Ocean [65] reported that management factors such as visitors allowed on farms and environmental mismanagement were associated with presence of ESBL/AmpC-producer *E*. *coli* on farms. The lack of significance may be due to the low statistical power related to our small sample size (n = 32 farms). It should be noted that our study was conducted during a period when poultry farms were generally rare. Indeed, some breeders, especially those in sector 2, are very irregular, producing only in times of high demand that correspond to the end-of-year celebrations [20]. This study could therefore provide the basis for a future study of a larger sample size, during a period of high demand, focussing on the potential risk factors that demonstrated a tendency to be associated with the presence of 3GC-resistant isolates on farms.

## Conclusions

*Escherichia coli* isolates potentially pathogenic for humans and demonstrating MDR, with resistance expressed against antimicrobials of critical importance in human health, were found in healthy chickens in Senegal. 3GC-resistance was due to the CTX-M/CMY-2 genes whereas ciprofloxacin-resistance was due to mutation in *gyrA* and *parC* genes of QRDR. Some clonally related ESBL/AmpC-producer isolates were observed, suggesting their circulation among farms. Moreover, the involvement of plasmids in the dissemination of ESBL/AmpC genes may be possible since plasmids harboring these genes were encountered in isolates of different clonal groups originating from different farms. These results highlight the importance of controlling antimicrobial use in Senegal and implementing surveillance system for AMR. This could include a network for AMR surveillance in veterinary medicine, control of the distribution of antimicrobials, combat the illegal sale of antimicrobials (black markets), sensitize breeders about the danger of self-medication, sensitize the veterinarians on the prior performance of the antibiogram before their use for therapeutic purposes and finally, fight against counterfeit medicines.

## Supporting information

S1 Fig Methodological approach used in this study. * For 3 faecal samples, 11 drinking water samples, 4 of rinsing water samples and 2 swabs, no lactose + colony growth on MacConkey agar. These samples were then inoculated into TSB + bile salt broths to remove bacteria other than *E*. *coli*, but no culture could be obtained. ** From 1 faecal sample and 2 drinking water samples, only one *uidA*+ isolate was found.
(PDF)

S1 Table PCR primers used for screening and identification of *bla*_*CTX-M*_ subtypes. (DOC)

S2 Table Prevalence of nonsusceptibility at the sample and farm levels in potential ESBL/AmpC-producer *Escherichia coli* from healthy chickens in the region of Dakar, Senegal. ^a^Category of human antimicrobials importance according to the World Health Organization (WHO) [37]: (I) Very High Importance, (II) High Importance, (III) Moderate Importance. ^b^Antimicrobial classes: (FLQ) Fluoroquinolones; (PEN/I) Penicillin+β-Lactamase inhibitors; (CPS) Cephalosporins; (PEN) Penicillin; (CPM) Cephamycin; (AMG) Aminoglycosides; (FOL) Folate inhibitors; (PHE) Phenicols; (TET) Tetracyclines. ^c^Antimicrobials: (NAL) Nalidixic acid; (CIP) Ciprofloxacin; (AMC) Amoxicillin/clavulanic acid; (TIO) Ceftiofur; (CRO) Ceftriaxone; (AMP) Ampicillin; (FOX) Cefoxitin; (GEN) Gentamicin; (KAN) Kanamycin; (STR) Streptomycin; (SXT) Trimethoprim-sulphamethoxazole; (SSS) Sulfisoxazole; (CHL) Chloramphenicol; (TET) Tetracycline.
(DOC)

S3 Table Prevalence of AMR genes by samples of origin. AMR, antimicrobial resistance; No., Number; %, prevalence expressed in percentage; Tetracycline resistance gene *tetC* was not detected in any isolate.
(DOC)
