# Supplementary figures and images for: Prevalence of antimicrobial resistance and potential pathogenicity, and possible spread of third generation cephalosporin resistance, in Escherichia coli isolated from healthy chicken farms in the region of Dakar, Senegal
Source: PLoS One. 2019 Mar 26;14(3):e0214304. doi: 10.1371/journal.pone.0214304 (PMC6435184; doi:10.1371/journal.pone.0214304)

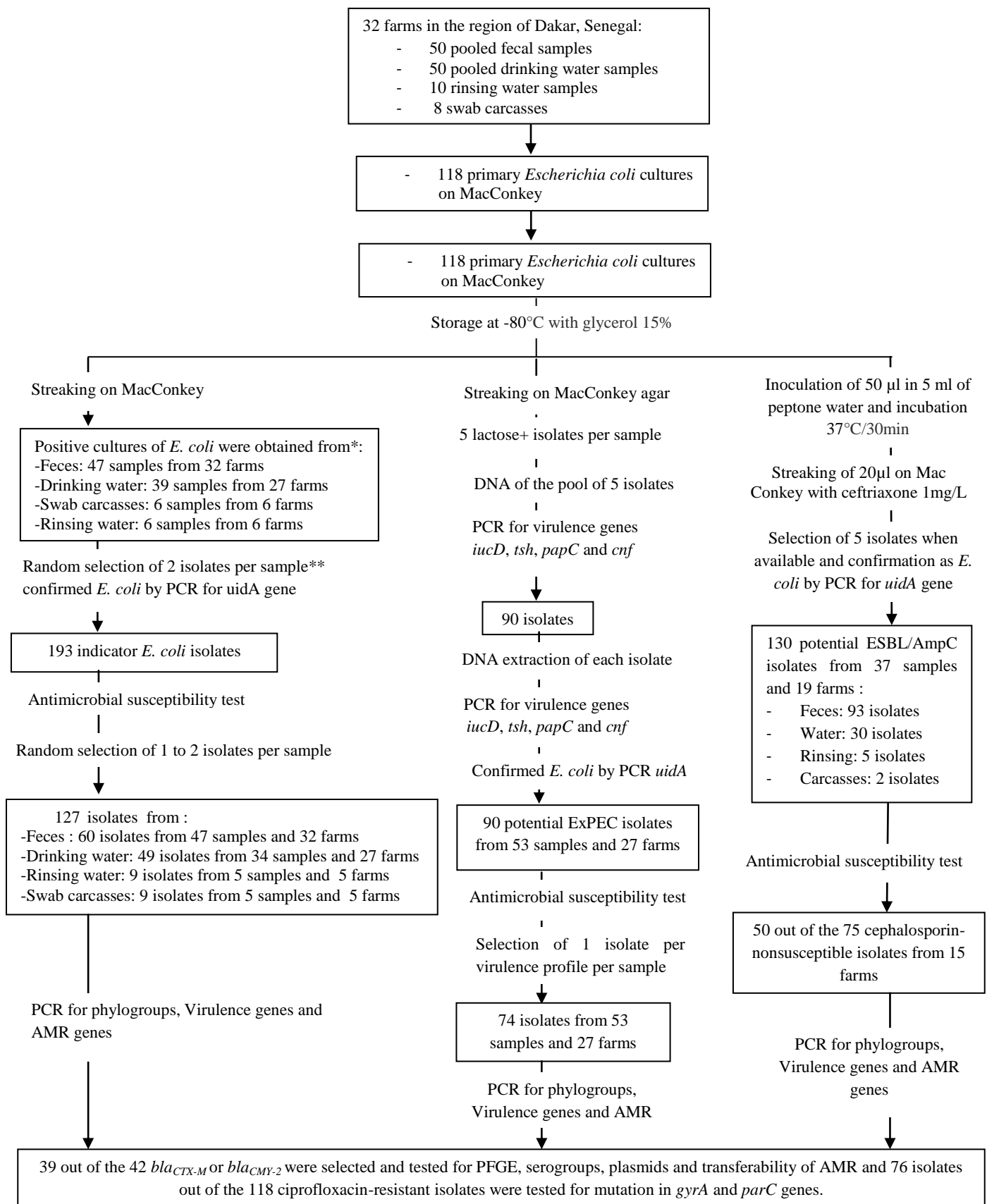

Supplement: S1 Fig — * For 3 faecal samples, 11 drinking water samples, 4 of rinsing water samples and 2 swabs, no lactose + colony growth on MacConkey agar. These samples were then inoculated into TSB + bile salt broths to remove bacteria other than E. coli, but no culture could be obtained. ** From 1 faecal sample and 2 drinking water samples, only one uidA+ isolate was found. (PDF) [file pone.0214304.s001.pdf]
