# Supplementary material for: Prevalence of antimicrobial resistance and potential pathogenicity, and possible spread of third generation cephalosporin resistance, in Escherichia coli isolated from healthy chicken farms in the region of Dakar, Senegal
Source: PLoS One. 2019 Mar 26;14(3):e0214304. doi: 10.1371/journal.pone.0214304 (PMC6435184; doi:10.1371/journal.pone.0214304)
Supplement: S1 Table — (DOC) [file pone.0214304.s002.doc]

**Table S1.** **PCR primers used for screening and identification of *bla_CTX-M_*subtypes.**

| **Product or antimicrobial** | **Gene** | **Primer** | **Amplicon size (bp)** | **Annealing temperature** | **Control strain** | **Reference** |
| --- | --- | --- | --- | --- | --- | --- |
| Ceftriaxone | *bla_CTX-M_* group 1 | 5’ TTCGTCTCTTCCAGAATAAGG 3’  5’ CAGCACTTTTGCCGTCTAAG 3’ | 968 | 55°C | 18772 | (Borgogna et al., 2016) |
| Ceftriaxone | *bla_CTX-M_* group 2 | 5’ CGTTAACGGCACGATGAC 3’  5’ CGATATCGTTGGTGGTRCCAT 3’ | 404 | 55°C | 18771 | (Borgogna et al., 2016) |
| Ceftriaxone | *bla_CTX-M_* group 8/25 | 5’ AACRCRCAGACGCTCTAC 3’  5’ TCGAGCCGGAASGTGTYAT 3’ | 326 | 55°C | 18773 | (Borgogna et al., 2016) |
| Ceftriaxone | *bla_CTX-M_* group 9 | 5’ TGGTGACAAAGAGAGTGCAACG 3’  5’ CACAGGCCTTCGGCGAT 3’ | 875 | 55°C | 18774 | (Paauw et al., 2006) |

**References**

Borgogna TR, Borgogna JL, Mielke JA, Brown CJ, Top EM, Botts RT, Cummings DE. High Diversity of CTX-M Extended-Spectrum β-Lactamases in Municipal Wastewater and Urban Wetlands. Microb Drug Resist 2016; 22: 312–320.

Paauw A, Fluit AC, Verhoef J, Leverstein-van Hall MA. *Enterobacter cloacae* Outbreak and Emergence of Quinolone Resistance Gene in Dutch Hospital. Emerg Infect Dis 2006; 12: 807–812.
