# Supplementary material for: Prevalence of antimicrobial resistance and potential pathogenicity, and possible spread of third generation cephalosporin resistance, in Escherichia coli isolated from healthy chicken farms in the region of Dakar, Senegal
Source: PLoS One. 2019 Mar 26;14(3):e0214304. doi: 10.1371/journal.pone.0214304 (PMC6435184; doi:10.1371/journal.pone.0214304)
Supplement: S2 Table — aCategory of human antimicrobials importance according to the World Health Organization (WHO) [37]: (I) Very High Importance, (II) High Importance, (III) Moderate Importance. bAntimicrobial classes: (FLQ) Fluoroquinolones; (PEN/I) Penicillin+β-Lactamase inhibitors; (CPS) Cephalosporins; (PEN) Penicillin; (CPM) Cephamycin; (AMG) Aminoglycosides; (FOL) Folate inhibitors; (PHE) Phenicols; (TET) Tetracyclines. cAntimicrobials: (NAL) Nalidixic acid; (CIP) Ciprofloxacin; (AMC) Amoxicillin/clavulanic acid; (TIO) Ceftiofur; (CRO) Ceftriaxone; (AMP) Ampicillin; (FOX) Cefoxitin; (GEN) Gentamicin; (KAN) Kanamycin; (STR) Streptomycin; (SXT) Trimethoprim-sulphamethoxazole; (SSS) Sulfisoxazole; (CHL) Chloramphenicol; (TET) Tetracycline. (DOC) [file pone.0214304.s003.doc]

**S2 Table.** **Prevalence of nonsusceptibility at the sample and farm levels in potential ESBL/AmpC-producer *Escherichia coli* from healthy chickens in the region of Dakar, Senegal.**

| Unit of study (No. examined) | Percentage (%) of units with one or more nonsusceptible isolates per category^a^, antimicrobial class^b^ and antimicrobial^c^ | | | | | | | | | | | | | |
| --- | --- | --- | --- | --- | --- | --- | --- | --- | --- | --- | --- | --- | --- | --- |
|  | Critically important | | | | | | | | | Highly important | | | | |
|  | Highest priority | | | | High priority | | | | |  |  |  |  |  |
|  | FLQ | | CPS | | PEN | PEN/I | AMG | | | CPM | FOL | | PHE | TET |
|  | NAL | CIP | TIO | CRO | AMP | AMC | GEN | KAN | STR | FOX | SXT | SSS | CHL | TET |
| Drinking water/Faeces (n=123) | 54.5 | 28.5 | 58.5 | 58.5 | 100 | 43.1 | 8.1 | 20.3 | 26.8 | 43.9 | 40.6 | 77.2 | 34.1 | 95.1 |
| Carcasses/rinsing water (n=7) | 100 | 85.7 | 42.8 | 42.8 | 100 | 14.3 | 0.0 | 57.1 | 42.8 | 14.3 | 85.7 | 85.7 | 14.3 | 71.4 |
| All sources (n=130) | 56.9 | 31.5 | 58.5 | 58.5 | 100 | 41.5 | 7.9 | 22.3 | 27.7 | 42.3 | 43.1 | 77.7 | 33.1 | 93.8 |
| Farms (n = 18) | 100 | 44.4 | 77.8 | 77.8 | 100 | 72.2 | 16.7 | 44.4 | 55.6 | 72.2 | 72.2 | 94.4 | 50.0 | 100 |

^a^Category of human antimicrobial importance according to the World Health Organization (WHO) [37].

^b^Antimicrobial classes: (FLQ) Fluoroquinolones; (PEN/I) Penicillin+β-Lactamase inhibitors; (CPS) Cephalosporines; (AMG) Aminoglycosides; (CPM) Cephamycin; (PEN) Penicillin; (FOL) Folate inhibitors; (PHE) Phenicols; (TET) Tetracyclines.

^c^Antimicrobials: NAL, Nalidixic acid; CIP, Ciprofloxacin; AMC, Amoxicillin/clavulanic acid; TIO, Ceftiofur; CRO, Ceftriaxone; AMP, Ampicillin; FOX, Cefoxitin; GEN, Gentamicin; KAN, Kanamycin; STR, Streptomycin; SXT, Trimethoprim-sulphamethoxazole; SSS, Sulfisoxazole; CHL, Chloramphenicol; TET, Tetracycline.

**References**

37. WHO [World Health Organization]. Antimicrobial Resistance: Global report on Surveillance. Available from : http://www.who.int/drugresistance/documents/surveillancereport/en/.
