# Supplementary material for: Prevalence of antimicrobial resistance and potential pathogenicity, and possible spread of third generation cephalosporin resistance, in Escherichia coli isolated from healthy chicken farms in the region of Dakar, Senegal
Source: PLoS One. 2019 Mar 26;14(3):e0214304. doi: 10.1371/journal.pone.0214304 (PMC6435184; doi:10.1371/journal.pone.0214304)
Supplement: S3 Table — AMR, antimicrobial resistance; No., Number; %, prevalence expressed in percentage; Tetracycline resistance gene tetC was not detected in any isolate. (DOC) [file pone.0214304.s004.doc]

**S3Table.** **Prevalence of AMR genes by samples of origin.**

| AMR gene | No. (%) of positive isolates by types of samples | | | |
| --- | --- | --- | --- | --- |
|  | Faeces (60) | Drinking water (n=49) | Rinsing water (n=9) | Carcasses (n=9) |
| *tetA* | 36 (60.0) | 27 (55.1) | 4 (44.4) | 6 (66.7) |
| *tetB* | 13 (21.7) | 7 (14.3) | 1 (11.1) | 4 (44.4) |
| *dfrA1* | 22 (36.7) | 10 (20.4) | 1 (11.1) | 4 (44.4) |
| *dfrA5* | 6 (10.0) | 7 (14.3) | 0 (0.0) | 0 (0.0) |
| *dfrA7* | 34 (56.7) | 21 (42.8) | 2 (22.2) | 4 (44.4) |
| *qnrB* | 4 (6.7) | 8 (16.3) | 1 (11.1) | 1 (11.1) |
| *aadA1* | 17 (28.3) | 3 (6.1) | 2 (22.2) | 3 (33.3) |
| *bla_TEM_* | 17 (28.3) | 20 (40.8) | 1 (11.1) | 8 (88.9) |
| *bla_OXA-1_* | 0 (0.0) | 1 (2.0) | 0 (0.0) | 0 (0.0) |
| *bla_SHV_* | 0 (0.0) | 2 (4.1) | 0 (0.0) | 0 (0.0) |
| *bla_CTX-M_* | 2 (3.3) | 0 (0.0) | 0 (0.0) | 0 (0.0) |
| *bla_CMY-2_* | 0 (0.0) | 1 (2.0) | 0 (0.0) | 0 (0.0) |
